# The effect of including a mobile arch, toe joint, and joint coupling on predictive neuromuscular simulations of human walking

**DOI:** 10.1038/s41598-024-65258-z

**Published:** 2024-06-27

**Authors:** Alexandra Buchmann, Simon Wenzler, Lauren Welte, Daniel Renjewski

**Affiliations:** 1 Chair of Applied Mechanics, Technical University of Munich, 85748 Garching, Germany; 2 Department of Mechanical Engineering, University of Alberta, Edmonton, AB T6G 2R3 Canada

**Keywords:** Computational biology and bioinformatics, Engineering, Mathematics and computing

## Abstract

Predictive neuromuscular simulations are a powerful tool for studying the biomechanics of human walking, and deriving design criteria for technical devices like prostheses or biorobots. Good agreement between simulation and human data is essential for transferability to the real world. The human foot is often modeled with a single rigid element, but knowledge of how the foot model affects gait prediction is limited. Standardized procedures for selecting appropriate foot models are lacking. We performed 2D predictive neuromuscular simulations with six different foot models of increasing complexity to answer two questions: *What is the effect of a mobile arch, a toe joint, and the coupling of toe and arch motion through the plantar fascia on gait prediction?* and *How much of the foot’s anatomy do we need to model to predict sagittal plane walking kinematics and kinetics in good agreement with human data?* We found that the foot model had a significant impact on ankle kinematics during terminal stance, push-off, and toe and arch kinematics. When focusing only on hip and knee kinematics, rigid foot models are sufficient. We hope our findings will help guide the community in modeling the human foot according to specific research goals and improve neuromuscular simulation accuracy.

## Introduction

Predictive neuromuscular simulations are a powerful tool for studying the biomechanics of human gait, allowing the investigation of *what-if* scenarios, such as the effect of altered muscle properties or different skeletal geometries on human gait, that would be impossible to study in living subjects^1,2^. Simulations can thus be used to advance the fundamental understanding of human biomechanics, and derive functional design criteria for technical devices such as prostheses or biorobots like EcoWalker^3^.

Important factors for accurate gait prediction include musculoskeletal geometry, neural control architecture^4^, muscle models^5^, and cost functions used in the optimization process^6,7^. Unfortunately, the importance of the foot model is often overlooked^2^. The correct representation of contact mechanics is essential in forward dynamics^8^, as contact forces directly affect system dynamics at the acceleration level. The foot is the terminal element of the human body which comes in contact with the ground. With 26 bones, more than a hundred muscles, tendons and ligaments^9^ and five primary axes of movement^10^, the anatomy of the foot is quite complex. Many neuromuscular simulation studies drastically simplify the anatomy of the human foot. Most use a completely rigid foot^4,6,7,11–13^ or a rigid arch with a toe segment^14–17^. A few studies have modeled a mobile arch with a toe segment, ligamentous structures^18,19^ or active foot muscles^20,21^.

**Figure 1:**
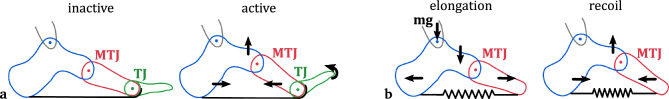
Illustration of windlass mechanism and arch spring theory. (**a**) Windlass mechanism as described in^24^. As the toe undergoes dorsiflexion, the PF wraps around the metatarsal head (green radius), causing the arch to shorten and rise. (**b**) Arch spring as described in^25^. Body weight applied to the foot elongates the arch, stretching the elastic ligaments. When the load is released, the arch recoils. Both explain the behavior of the human foot arch during walking. The illustrations were inspired by^23^. The windlass theory requires the PF to be a rather stiff structure, while the arch spring theory is based on the assumption that the PF is elastic.

The foot arch plays a critical role in stance and push-off. Ligaments and bony support^22^ passively maintain the shape of the arch. At the beginning of stance, the arch lengthens due to the applied body weight, while at the end of stance, it shortens for push-off. The arch spring and windlass mechanisms, illustrated in Fig. 1, are two theories that explain the motion and elastic properties of the arch during stance and push-off^23^. On the one hand, the windlass mechanism assumes a longitudinally stiff plantar fascia (PF). In late stance, dorsiflexion of the toe causes the PF to wrap around the metatarsal head. The resulting shortening of the PF and raising of the arch^24^ couples the motion of the toe joint (TJ) and midtarsal joint (MTJ). The arch spring hypothesis, on the other hand, assumes elastic properties of the PF and plantar ligament (PL). During early and mid-stance, the arch is compressed vertically and elongated horizontally by body weight. The elongation stretches the elastic ligaments that store energy. At the end of stance, the arch recoils, and the resulting energy release assists push-off^25^. Both theories have merit, as the PF and PL have elastic properties, and the PF couples toe motion to arch mobility.

Knowledge of how arch mobility, TJ motion, and their coupling via the PF influence gait prediction in neuromuscular simulations is still limited^20,26^, and a standardized process for formulating models of the human foot is lacking^26,27^. Adding a toe to a stiff foot was found to enhance the consistency between simulation and human data for knee stance kinematics, knee extension torques, and muscle activity, but worsened the consistency for ankle kinematics^17^. Based on Falisse et al.^17^, DHondt et al.^20^ developed a three-segment foot model with a mobile arch, plantar ligaments, and active foot muscles. They showed improved gait prediction compared to one- and two-segment foot models, as well as increased push-off power. Song et al.^21^ showed that walking with feet with a flexible arch and TJ makes the neuromuscular model less efficient compared to a rigid foot model.

To the best of our knowledge, no study has systematically compared and analyzed how a mobile arch, toe joint motion, and the interaction between the two influence gait predictions for 2D generic reflex-based neuromuscular simulations. Our study aims to fill this gap by first answering: **(I) What is the effect of a mobile arch, a toe joint, and the coupling of toe and arch motion through the PF on gait prediction for neuromuscular simulations?** Second, based on our results, we answer: **(II) How much of the foot’s anatomy do we need to model to predict sagittal plane walking kinematics and kinetics with neuromuscular simulations in good agreement with human data?**

We compare the simulation results with human reference data for ground reaction forces (GRFs) and joint kinematics for the hip, knee, and ankle. Additionally, we compare trends within the simulations for energy dissipation during contact, walking speed, and ankle push-off as essential characteristics for energy efficient walking, and natural leg dynamics^28^. To separate the effects of arch mobility, toe motion, and joint coupling, we analyze six different foot models on a 2D generic neuromuscular simulation actuated by Hill-type muscles with neural reflex control^4^. We start with a flat, rigid foot model and gradually increase the mechanical complexity up to a model with mobile arch and toe joint coupled by the PF. We modified only the kinematic foot structure while keeping the rest of the simulation unchanged, including its dimensions, mechanical structure, muscles, and neural control architecture. Based on our findings, we provide recommendations on which foot models to use for different simulation applications.

## Methods

### Foot models

**Figure 2:**
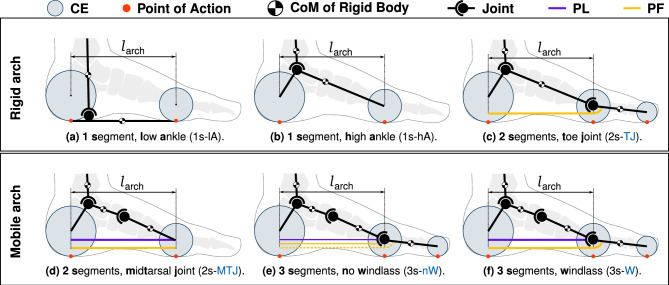
Overview of the six implemented foot models. All models share the same contact element geometries and mathematical contact formulation in the horizontal^29^ and vertical^4^ directions. The points of action where GRFs are applied are marked with a red dot. Models with a MTJ or a TJ include a PL and/or the PF. With and without windlass configuration, the PF force is calculated differently, resulting in coupling or decoupling between TJ and MTJ. The undeformed distance between the center of the heel and the ball contact point $l_{\text {arch}} = 17\,\hbox {cm}$ is kept constant for all foot models.

**Figure 3:**
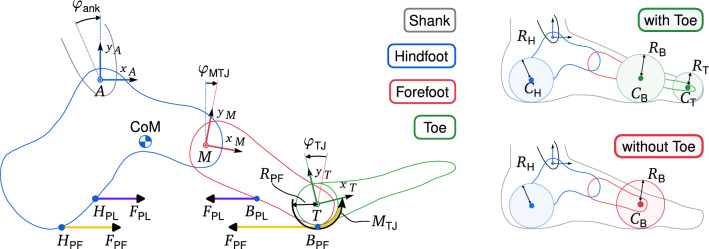
Model details with nomenclature, coordinate systems, and angular conventions. Hindfoot, forefoot and toes with their local coordinate systems *A*, *M* and *T* are modeled as rigid bodies. Both, $m_\text {foot}$ and $I_\text {foot}$ are defined at the center of mass, which is fixed relative to the hindfoot. Forefoot and toes are mass-less to ensure that all foot models have the same inertial properties. Table 1 lists the locations of all coordinate systems as well as the foot’s total mass and inertia. According to conventions in our reference literature^32,33^, the angles $\varphi _\text {ank}$ and $\varphi _\text {MTJ}$ are defined as clockwise rotations around the z-axis between the shank coordinate system and *A* and between *A* and *M*, respectively. $\varphi _\text {TJ}$ is defined as a counter-clockwise rotation between *M* and *T*. To return the foot to its neutral position during swing and to ensure numerical stability, the MTJ and TJ contain linear, rotational spring-damper elements (not shown) with $k=0.01\,\textrm{Nm}/{\deg}$ and $d=0.001\,\textrm{Nms}/{\deg}$. Their influence during stance is negligible, as the resulting torques and damping are very small compared to the effects of the ligaments (torque at max. flexion: 0.69 Nm). The heel, ball, and toe contact elements are named $C_H$, $C_B$, and $C_T$, respectively. For foot models with a toe segment, $C_B$ is attached to the toe segment to allow relative motion between the forefoot and toe without rotating the contact element at the ball; for foot models without toes, $C_B$ is attached to the forefoot.

We implemented six different foot models shown in Fig. 2 to test them on a 2D, reflex-controlled neuromuscular simulation^4^ in Matlab Simulink R2020b (The Mathworks Inc., Natick, Massachusetts, USA). The models are divided into rigid and mobile arch models. Model nomenclature in the text follows the systematic format: *# segments - special feature*. Details on mechanical segment properties, joint locations and model dimensions are given in Fig. 3 and Table 1. **1s-lA** is a simple rigid body with two circular contact elements at the heel and ball. The undeformed distance between the centers of the heel and ball contact spheres $l_\text {arch}$ is the same for all models. For **1s-hA** we introduce a high ankle joint which effectively lowers the contact elements with respect to the ankle, resembling a rigid arch. Finally, we add a toe to this rigid arch to obtain **2s-TJ**. The stiffness of the toe joint is provided by the PF which wraps around the metatarsal head, modeled with radius $R_\text {PF}$. Bending the toe thus stretches the elastic PF and induces an angle-dependent joint moment.

For mobile arch models, the MTJ is inserted between the hindfoot and midfoot. The arch is spanned by PL and PF to keep it lifted, see **2s-MTJ** in Fig. 2. We add a toe to this mobile arch as we did for the rigid arch models. Since the PF now spans the mobile arch with the MTJ and the toe joint, we get a coupling between arch motion and toe motion. During toe dorsiflexion, the rather stiff PF wraps around the metatarsal head and shortens the arch, resulting in plantarflexion of the MTJ, which resembles the windlass (W) mechanism in **3s-W**.

Since we want to systematically investigate the effect of adding a toe to a mobile arch versus coupling toe and arch motion, we also look at how the fictional model **3s-no-windlass (nW)** without coupling between MTJ and TJ behaves. The toe joint moment generated by the PF stretch is calculated as if the arch did not move, while the force of the PF on the arch is calculated as if the toe joint did not move.

### Ligaments

Arch elasticity and toe joint stiffness are modeled by two elastic ligaments, the PL and the PF. We used the constitutive equation for the PF proposed by^30^ and added damping:

1 $$\begin{aligned} F(\lambda , \dot{\lambda })={\left\{ \begin{array}{ll} A \lambda ^2 \frac{k}{2 \alpha }\{\exp [\alpha (\lambda ^2-1)]-1\}\{1+d \dot{\lambda }\},&{} \text {if } \lambda \ge 1 \text { and } d\lambda > -1\\ 0, &{} \text {otherwise.} \end{array}\right. } \end{aligned}$$

where $\lambda (l)=l/l_0$ is the strain ratio of the current ligament length *l* and the resting length $l_0$. *A* is the cross-sectional area of the ligament, *k* models the stiffness of the undeformed collagen fibers, *d* is the damping parameter, and $\alpha$ is a dimensionless constitutive parameter related to the nonlinear elastic response of the tissue. The conditions $\lambda \ge 1 \text { and } d\lambda > -1$ ensure that the ligaments can only exert tensile forces when stretched and the maximum relaxation velocity $d\lambda$ is not exceeded.

We chose a very small damping parameter for $d_\text {PF}$ to avoid numerical oscillations, since the PF is described as an elastic structure that returns most of its stored energy^25^. The PL captures the residual stiffness and damping of the numerous plantar ligaments and muscles in the human arch in addition to the PF^25,31^. $d_\text {PL}$ was therefore chosen significantly higher. $l_0$ for both ligaments is defined by the foot geometry and the metatarsal head radius $R_\text {PF}$. Stiffness and cross-sectional area for PF were taken from^30^. To tune the cross-sectional area $A_\text {PL}$, we implemented a virtual test bench that emulates the in vivo experiment of^23^, testing the static compression behavior of the foot models under body weight. Assuming the same collagen fiber stiffness *k* for PF and PL, we tuned $A_\text {PL}$ so that the static arch compression and MTJ angle compared well with the experimental data from^23^ (see supplements Table S2 for details). All parameter values are listed in Table 1.

The length of PL is calculated as $l_{PL} = ||\textbf{r}_{H_\text {PL}} - \textbf{r}_{B_\text {PL}}||$ where $\textbf{r}_{H_\text {PL} }$ and $\textbf{r}_{B_\text {PL}}$ are the vectors to the heel and ball attachment points, respectively. The length of PF is calculated differently for each foot model, depending on whether there is a toe and whether the TJ and MTJ are coupled or decoupled:

**Table:** 

**2s-MTJ**:	**2s-TJ**:	**3s-nW**:	**3s-W**:
$l_\text {PF} = ||\textbf{r}_{H_\text {PF}} - \textbf{r}_{B_\text {PF}}||,$	$l_\text {PF} = l_0 + \varphi _\text {TJ} R_\text {PF},$	$l_\text {PF,1} = ||\textbf{r}_{H_\text {PF}} - \textbf{r}_{B_\text {PF}}||,$ $l_\text {PF,2} = l_0 + \varphi _\text {TJ} R_\text {PF},$	$l_\text {PF} = ||\textbf{r}_{H_\text {PF}} - \textbf{r}_{B_\text {PF}}|| + \varphi _\text {TJ} R_\text {PF},$

where $\textbf{r}_{H_\text {PF}}$ and $\textbf{r}_{B_\text {PF}}$ are the vectors to the ligament attachment points at heel and ball, $\varphi _\text {TJ}$ is the TJ angle, $R_\text {PF}$ is the radius of the metatarsal head, and $l_0$ is the ligament resting length. For the rigid arch in **2s-TJ**, the distance between the attachment points $H_\text {PF}$ and $B_\text {PF}$ is constant (see Fig. 3) and equal to the resting length $l_0$. For **3s-nW**, $F_\text {PF,1}$ acting on the attachment points $H_\text {PF}$ and $B_\text {PF}$ is calculated with $l_\text {PF,1}$ as if the toe would not move. Second, the moment $M_\text {TJ}$ at the toe joint is calculated with $l_\text {PF,2}$ as if the MTJ would not move. In **3s-W**, the TJ and MTJ motions are coupled.

**Table 1:** Geometric specifications, ligament, ground contact and foot parameters.

Geometrical model setup				Contact model			Ligaments, foot mass and inertia			
Vector	Unit	**1s-lA**	All others	Param.	Unit	Value	Param.	Unit	PF	PL
${}_{A}{\varvec{r}}_{M}$	cm	[7, − 1.4]		$k_y$	kN/m	78.30	*A*	$\hbox {mm}^{2}$	50	120
${}_{M}{\varvec{r}}_{T}$	cm	[6, − 4.6]		$v_\text {max}$	m/s	0.03	*k*	MPa	340	340
${}_{A}{\varvec{r}}_\text {CoM}$	cm	[5, − 5]		$\mu _d$	–	0.80	*d*	s	3	30
${}_{A}{\varvec{r}}_{H_\text {PL}; H_\text {PF}}$	cm	[− 2, − 6]		$\mu _s$	–	0.90	$l_0$	cm	15	15
${}_{M}{\varvec{r}}_{B_\text {PL}; B_\text {PF}}$	cm	[− 2, − 6]		$v_t$	m/s	0.01	$\alpha$	–	9	9
${}_{A}{\varvec{r}}_{C_H}$	cm	[− 4, 5]	[− 4, − 3]	$R_B$	cm	5.73	$R_\text {PF}$	cm	1	1
${}_{A}{\varvec{r}}_{C_B}$	cm	[13, 5.73]	[13, − 2.27]	$R_H$	cm	5.00	$m_\text {foot}$	kg	1.160	
${}_{A}{\varvec{r}}_{C_T}$	cm	–	[18.5, 0]	$R_T$	cm	0	$I_\text {foot}$	$\hbox {kg m}^{2}$	0.005	

**Geometric model setup:** Fig. 3 visualizes the model geometry. ${}_{A}{\varvec{r}}_{B}$ denotes the vector [x, y] from the origin of the coordinate system A to point B expressed in A. The horizontal positions of the contact elements, the height of the arch and the position of the TJ are based on anthropometric data^18^. Contrary to^18^, our MTJ is not aligned with the ankle joint, but is in its physiological position^23^, Fig. 6,^34^. **Contact Model:** Contact stiffness $k_y$, maximum relaxation speed $v_\text {max}$, dynamic and static friction coefficients $\mu _d$ and $\mu _s$ and transition velocity $v_t$ for Eqs. 2 and 3 to calculate normal^4^ and tangential^29^ contact forces. $R_{H;\,B;\,T}$ are the radii of the circular contact elements for heel, ball and toe based on^13,15^. **Ligaments, Foot Mass and Inertia:** Ligament cross-sectional area *A*, stiffness *k*, damping *d* and dimensionless constitutive parameter $\alpha$ for Eq. 1. The ligament properties *k* and $\alpha$ for PF and PL are taken from^30^, the cross-sectional area $A_\text {PF}$ for PF from^35^. Foot mass $m_\text {foot}$, center of mass and inertia $I_\text {foot}$ are based on anthropometric data from^36,37^. Mass and inertia are defined directly at the center of mass, which is fixed relative to the hindfoot. The forefoot and toes are modeled massless to ensure that all foot models have the same inertial properties.

### Contact model

The ground contact is modeled using circular contact elements with different radii for heel, ball and toe (see Fig. 3). The vertical and horizontal GRFs $F_v$ and $F_h$ are applied to the contact elements at their point of action, which is the point on the surface of the circular contact element with the deepest penetration with respect to the ground. As the contact elements rotate, their point of action moves in a rolling motion along their circumference. The radius of the heel contact element $R_{H} = 5\,\textrm{cm}$ is based on geometrically optimized models from^13,15^. $R_{B} = 5.73\,\textrm{cm}$ was chosen so that models without toes have the same effective foot length at roll-off as models with toes. Using our given toe geometry, we designed $R_B$ so that after $50^{\circ }$ rotation of the contact element, the point of action would be at the same point as the toe tip of a model with a toe. Preliminary tests showed no significance for the toe contact element radius $R_{T}$, which was then set to zero, i.e. a point contact.

The vertical contact force $F_v$ is calculated using a Hunt and Crossley viscoelastic contact formulation from^4^ inspired by^18,38^:

2 $$\begin{aligned} F_v= {\left\{ \begin{array}{ll} -k_y\Delta y(1-\bar{v}_y) &{} \text {if}\quad \Delta y\le 0\quad \text {and}\quad \bar{v}_y < 1\\ 0 &{} \text {otherwise,} \end{array}\right. } \end{aligned}$$

with contact stiffness $k_y$ and vertical penetration depth $\Delta y$ measured from the ground to the point of action. The vertical velocity $v_y$ of the contact point is normalized by the maximum relaxation velocity $v_{max}$ to $\bar{v}_y = v_y/v_{max}$. If $v_y$ exceeds $v_{max}$, i.e. the foot moves up very fast, $F_v$ is set to zero.

A continuous velocity-based friction model from^29^ is used for horizontal contact forces:

3 $$\begin{aligned} F_h = F_v \left( \mu _d \tanh (4\bar{v}_x) + (\mu _s - \mu _d) \frac{\bar{v}_x}{\left( \frac{1}{4}\bar{v}_x^2+\frac{3}{4}\right) ^2} \right) , \end{aligned}$$

with the dynamic coefficient of friction $\mu _d$, the static coefficient of friction $\mu _s$, and the horizontal velocity $v_x$ normalized by the transition velocity $v_t = 0.01\,\textrm{m}/\textrm{s}$ to $\bar{v}_x = v_x/v_t$. Compared to the nonlinear spring damper model used in^4^, which is similar to Eq. 2, this formulation significantly increases computational efficiency because the function has no state transitions or discontinuities.

### Predictive neuromuscular simulation

We use a 2D reflex-controlled neuromuscular simulation from Geyer and Herr^4^ implemented in Matlab Simulink R2020b (The Mathworks Inc., Natick, Massachusetts, USA). The model in its original configuration consists of seven rigid bodies, a trunk and two legs with femur, tibia, and foot, connected by revolute joints. Seven muscle groups per leg, Gastrocnemius (GAS), Soleus (SOL), Vastus group (VAS), Tibialis anterior (TA), Gluteus group (GLU), Hip flexor muscles (HFL), and Hamstrings (HAM), actuate the system. For modeling details see^39^, Fig. 1(a), p. 23.

For each foot model we optimized the simulation for steady-state walking using the global optimization framework of^39^ with a multi-objective cost function, Eq. 4. Our cost function minimizes energy expenditure, muscle fatigue, upper body (head-arm-trunk segment (HAT)) acceleration, knee and ankle stop moments, and aims for a smooth GRFs with minimal changes in the GRF rate, i.e., the first derivative of the forces:

4 $$\begin{aligned} J= & {} \frac{1}{x_\text {walk}} \int _{t_\text {3rd step}}^{t_\text {sim, max}} \Bigg (\, \underbrace{w_1 \cdot E_\text {met} }_{\begin{array}{c} \text {metabolic} \\ \text {energy }{(W)} \end{array}} + \underbrace{w_2 \cdot ACT^2 }_{\begin{array}{c} \text {muscle}\\ \text {fatigue }{(-)} \end{array}} + \underbrace{w_3 \cdot {\left|{GRF_\text {rate}}\right|}}_{\begin{array}{c} \text {ground reaction}\\ \text {force rate}\,(\frac{\textrm{N}}{\textrm{s}}) \end{array}} + \underbrace{w_4 \cdot {\left|{a_\text {HAT}}\right|}}_{\begin{array}{c} \text {HAT} \\ \text {acceleration }{(\frac{\textrm{m}}{\textrm{s}^2})} \end{array}} + \cdots \nonumber \\{} & {} \cdots \, \underbrace{w_5 \cdot |T_\text {kne}| }_{\begin{array}{c} \text {stop moments} \\ \text {knee (Nm)} \end{array}} + \underbrace{w_6 \cdot |T_\text {ank}| }_{\begin{array}{c} \text {stop moments} \\ \text {ankle (Nm)} \end{array}} \,\Bigg ) dt \, + \underbrace{w_7 \cdot (t_\text {sim, max} - t_\text {sim, des})^2 }_{\begin{array}{c} \text {early fall} \\ \text {penalty (-)} \end{array}}. \end{aligned}$$

The model takes a few steps to settle into a steady state walk, so the cost function only considers measures from the 3rd step on. All measures are normalized to $x_\text {walk}$, the total distance the model walked from step three to the end of the simulation. If the model is not able to walk the full simulation time without falling, an early fall penalty is applied. The penalty is the difference between the desired total simulation time, $t_\text {sim, des}$, and the maximum simulation time reached when falling, $t_\text {sim, max}$. For stable walking this penalty term is zero.

Table 2 shows the cost function weights for Eq. 4 and Table 3 lists the optimization results obtained for all foot models. We optimize the nine muscle feedback gains $G_m$, and the knee hyperextension gain $k_\varphi$ of the reflex controller. The neural controller uses two main types of local feedback to compute the muscle stimulus $S_m(t)$ of a given muscle *m*. Force feedback, where $S_m(t) = S_{0,m} + G_m F_m (t - \Delta t_m)$ and length feedback where $S_m(t) = S_{0,m} + G_m l_{\text {CE}, m} (t - \Delta t_m)$, where $S_{0,m}$ is the resting stimulus, $\Delta t_m$ is a muscle-specific time delay, $F_m$ is the current muscle force, and $l_{\text {CE}, m}$ is the length of the contractile element of the muscle. Force feedback gains are several orders of magnitude smaller than length feedback gains and are normalized to the maximum muscle force $F_\text {max}$ as $S_m(t) \in [0,1]$, see Table 3. For details, see^4^.

The cost function has a strong emphasis on smooth GRFs, as this has been shown to result in good agreement with human data, especially for ankle push-off timing and GRF^6^. We solved the optimization problem using the genetic algorithm Matlab GA, Optimization Toolbox Version 9.0, Matlab R2020b (The Mathworks Inc., Natick, Massachusetts, USA)^40–42^ with a population size of 200 particles and a maximum of 50 stall generations as stopping criterion. Each simulation ran for 15 s simulation time and was solved with ode15s, Matlab R2020b (The Mathworks Inc., Natick, Massachusetts, USA)^43,44^ for stiff differential equations with a relative tolerance of $10^{-4}$ and a maximum step size of 0.1 s. We checked all optimizations for convergence within generations.

**Table 2:** Equation 4 cost function weights.

		No.	Value	Rel. (%)
$E_\text {met}$	(W)	$w_1$	$5.35\,\textrm{e}2$	$53.1 \pm 2.6$
$ACT^2$	(–)	$w_2$	$1\,\textrm{e}3$	$4.9 \pm 0.3$
$GRF_\text {rate}$	$\left( \frac{\textrm{N}}{\textrm{s}}\right)$	$w_3$	3	$18.6 \pm 1.1$
$a_\text {HAT}$	$\left( \frac{\textrm{m}}{\textrm{s}^2}\right)$	$w_4$	$1\,\textrm{e}4$	$18.3 \pm 1.2$
$T_{K}$	(Nm)	$w_5$	15	$3.7 \pm 1.2$
$T_{A}$	(Nm)	$w_6$	20	$1.1 \pm 0.2$
Pen.	(–)	$w_7$	$1\,\textrm{e}5$	0

*Rel.* gives the relative contribution of each term to the average final cost ($496.0 \pm 17.5$) with standard deviation.

**Table 3:** Optimization results.

Arch	Rigid			mobile		
Toe	None		Decpl.	None	Decpl.	Cpl.
Gain	1s-lA	1s-hA	2s-TJ	2s-MTJ	3s-nW	3s-W
$k_{\varphi }$	2.02	*2.94*	2.00	2.00	2.00	2.00
$G_{HAMHFL}$	*2.00*	*3.00*	4.00	*3.50*	*3.51*	*3.47*
$\mathbf {G_{GAS}}$	1.10	1.10	1.09	1.10	1.10	1.10
$G_{TA}$	*1.23*	1.10	*2.10*	*2.60*	1.07	1.10
$\mathbf {G_{VAS}}$	*1.24*	1.15	1.15	1.15	1.15	1.15
$\mathbf {G_{SOLTA}}$	*4.00* *e*3	*4.27*	*1.22*	*0.73*	0.30	*4.25* *e*3
$\mathbf {G_{SOL}}$	1.20	1.20	1.20	1.20	1.20	1.20
$G_{HFL}$	*0.41*	*0.38*	0.35	*0.29*	0.35	*0.85*
$\mathbf {G_{HAM}}$	0.65	0.65	0.65	0.65	0.65	0.65
$\mathbf {G_{GLU}}$	0.40	*0.49*	*0.46*	0.40	0.40	0.40

Force feedback gains of GAS, VAS, SOL, SOLTA, HAM, and GLU marked in bold are normalized to the muscle’s $F_{max}$. Gains that differ from^4^ by at least 5% of the nominal value are highlighted in italic. All model parameters not listed here are identical to^4^.

### Data analysis

All simulation results show the last full gait cycle of the left leg. The GRFs are filtered using a zero-phase Butterworth filter, Matlab R2020b (The Mathworks Inc., Natick, Massachusetts, USA)^45–47^ with a cutoff frequency of 50 Hz to remove the high-frequency, large initial load peak for plotting. A gait cycle is defined from heel strike to subsequent heel strike. Steps are detected based on the filtered vertical GRFs with a detection threshold of 1 N for the ball contact element. Human data for GRFs, range of motion, and joint power are from^32^, Trial 20, where participants walked at a fixed speed of 1.25 m/s with self-selected stride length and frequency. The raw data from^32^ are first averaged over five strides per participant and then averaged over all participants. We calculated the power amplification $P_\text {amp}$ listed in Table 4 according to^28^ to quantify the effectiveness of the ankle push-off. $P_\text {amp}$ is defined as the maximum positive power peak during push-off divided by the minimum negative peak during stance. Joint kinematics of TJ and MTJ are taken from^30^. The double-support phases in the plots are extracted from the human data, so they may not perfectly match the simulations. We also analyzed the normalized maximum cross-correlation for hip, knee, and ankle kinematics with human data for one stride. An R-value close to one indicates perfect agreement between the two signals, while a value close to zero indicates no agreement at all. Time is always normalized to stride time.

## Results

We assessed GRFs, energy dissipation during stance phase, joint kinematics of hip, knee, and ankle, ankle push-off mechanics and global gait measures including energy efficiency and walking speed of the six foot models presented. To identify the required level of modeling detail for individual simulation results, we analyzed the effects of a mobile arch, toes, and arch-toe coupling on these outcomes.

### Ground reaction forces, center of pressure and work

**Figure 4:**
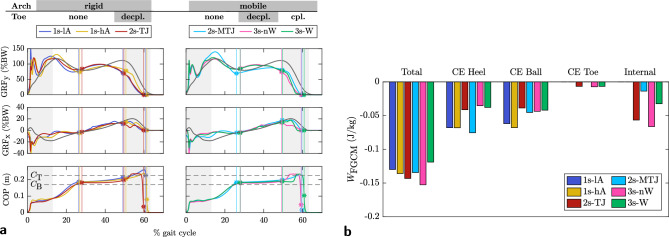
Vertical and horizontal GRFs, CoP progression and foot model work. (**a**) Vertical and horizontal GRFs and CoP progression within the foot with respect to the heel position at touch-down. (**b**) Net dissipative foot work with contributions of individual contact elements and internal foot work of PF and PL. Gray lines show human data from^48^, light gray areas mark corresponding double support phases. Vertical colored lines indicate heel-off, opposite touch-down, and toe-off. $C_B$ and $C_T$ denote the positions of the ball and toe contact elements with respect to the heel. The work of heel and ball contact elements is around $-0.040$ J/kg, the toe dissipate significantly less with $-0.007$ J/kg. Both, heel/ball and toe contact element dissipation compare well to^49^ with $-0.036$ J/kg and $-0.006$ J/kg, respectively. The internal dissipation captures damping effects in the PF and PL. 3s-nW has the highest internal dissipation of $-0.066$ J/kg. The total dissipative work of 0.126–0.152 J/kg for all elements in the foot is higher than what was reported in literature for walking, e.g.^49^ measured $-0.083$ J/kg ± 0.021 J/kg.

**Figure 5:**
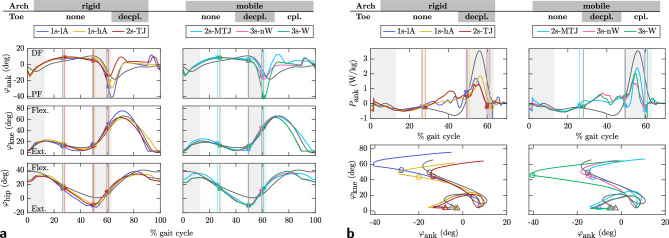
Joint kinematics and ankle performance curve. (**a**) Joint kinematics for ankle, knee and hip. Normalized maximum cross-correlation to human data is provided in Table S1. (**b**) Ankle joint power and knee-ankle coordination until beginning of swing phase. Triangles mark touch-down, circles toe-off. Gray lines show human data from^32^, light gray areas mark corresponding double support phases. Directions of motion are indicated by flexion (Flex.), extension (Ext.), dorsiflexion (DF), and plantarflexion (PF). Vertical colored lines mark heel-off, opposite touch-down, and toe-off for each model. Ankle kinematics and push-off power depend on the foot model, while knee and hip kinematics are not affected. 2s-MTJ shows the highest push-off peak with 2.4 W/kg while 2s-TJ exhibits the smallest peak with with 1.3 W/kg.

Figure 4a shows the vertical and horizontal GRFs, and the progression of the center of pressure (CoP) within the foot after touch-down. All models show an M-shaped double hump profile for the vertical GRFs. The first hump is in good agreement with human data, while the second hump is less pronounced and the peak occurs earlier. The opposite leg touch-down timing correlates well with human data. All models show a 2% shorter stance phase and early heel rise at about 25% stride, in contrast to human data at 30% stride^48^. Models with a toe exhibit their second peak 5% earlier than models without a toe and the first GRF peak is reduced by about 10 N. 2s-MTJ shows a remarkable initial load peak of 140% BW, which distinguishes it from all other models. For horizontal GRFs, the progression from breaking to propulsive forces is similar to human data. Including a TJ without coupling it to the MTJ (2s-TJ, 3s-nW) decreases positive horizontal GRFs at the end of stance and results in reduced forward propulsion, which is also reflected in reduced ankle push-off power, shown in Fig. 5b. 2s-MTJ with a mobile arch shows propulsive forces at the end of stance, similar to human data. The CoP progresses similarly for all models to the opposite leg’s touch-down. During double support, the CoP progresses rapidly to the tip of the toe for models with a decoupled toe (2s-TJ, 3s-nW), while the CoP remains under the toe until the maximum horizontal GRF is reached for the fully coupled model (3s-W)).

**Figure 6:**
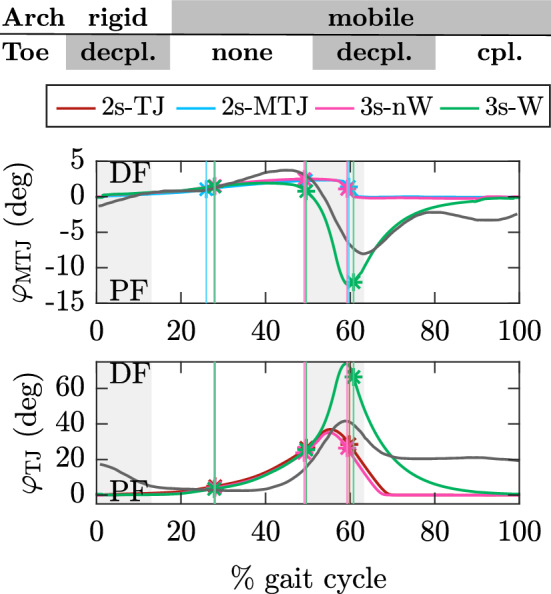
TJ and MTJ kinematics. Gray areas show human double support. Gray lines show human data^33^. Vertical lines mark heel-off, opposite touch-down, and toe-off. Please note that the reported range of motion for MTJ and TJ varies widely in the literature. (MTJ: $8^{\circ }$^51^ up to $17.6^{\circ }$^34^, TJ: $44.9^{\circ }$^33^ up to $64^{\circ }$^51^).

The total mechanical work of all foot models shown in Fig. 4b is negative, indicating dissipative behavior as expected. The heel and ball contact elements dissipate similar amounts of energy, while significantly less is dissipated at the toe. This is reasonable because the heel and ball experience higher impact velocities and forces than the toe. 2s-MTJ does behave different, the heel here dissipates more than the ball element. The ligaments dissipate energy due to damping effects in the constitutive Eq. 1 for PF and PL. Models with a decoupled toe (2s-TJ and 3s-nW) have the highest internal dissipation. The motion of the TJ here is the main reason for the stretching of the PF, while the MTJ motion of only $2^{\circ }$ shown in Fig. 6 contributes minimally. The coupled model (3s-W) shows a reduced total dissipation compared to the uncoupled model (3s-nW).

### Joint kinematics, ankle power and global gait measures

Knee and hip kinematics shown in Fig. 5a are similar for all models and correlate well with human data ($R_\text {hip, knee} \ge 0.94$). The upper body is generally more forward leaned in the simulation compared to human data, as seen in the 10–$15^{\circ }$ reduced hip extension at the beginning and end of stance. Ankle stance kinematics are similar across models until the opposite leg touches down. Ankle kinematics are less consistent with human data than knee and hip ($R_{\text {ank, stance}} = 0.82$) and show maximum dorsiflexion 20% earlier than human data due to the models’ early heel rise. At the onset of double support, ankle plantarflexion shows a large difference between the models ranging from 20 to $40^{\circ }$. Less plantarflexion occurs in the decoupled toe and mobile arch models (2s-TJ, 2s-MTJ and 3s-nW), while the fully rigid low foot (1s-lA) and the coupled model (3s-W) show the most plantarflexion. The presence of a decoupled toe (2s-TJ, 3s-nW) reduces the plantarflexion velocity, as seen in the slopes of the ankle angle plots in Fig. 5a. Toe-off always occurs before the maximum point of plantarflexion, which mainly affects the swing phase. Detailed normalized cross-correlation values for all models during swing and stance phase are provided in the supplementary material Table S1.

Ankle unloading with positive power output starts too early for all models due to the aforementioned early heel rise, as seen in Fig. 5b. The beginning of the high, impulsive late stance positive ankle power coincides with the opposite leg’s heel strike, similar to human data. The mobile arch without toes (2s-MTJ) shows the highest push-off peak followed by the fully coupled model with toes (3s-W). Introducing only a TJ without coupling it to the MTJ (2s-TJ, 3s-nW) results in a 45% reduction in maximum push-off power compared to the model with mobile arch only (2s-MTJ). Rigid models (1s-lA, 1s-hA) fall in between and have a less pronounced power peak than 2s-MTJ, but a reasonable power amplification compared to models with decoupled toes (2s-TJ, 3s-nW). Ankle push-off relies on the interplay between knee and ankle kinematics shown in Fig. 5b below the ankle power plot. Due to the early onset of ankle plantarflexion in the simulation, all models show a clockwise loop prior to push-off that is not present in the human data, reflecting the early unloading of the ankle between heel-off and opposite touch-down. In the human data, push-off begins when the ankle is in maximum dorsiflexion and the knee begins to flex. In the simulations, it begins while the knee is still extending in mid-stance, before the large power peak occurs at a similar time as in humans.

Table 4 summarizes the global gait measures. The duty factor, i.e. the relative contribution of stance time to the total stride time, remains constant. Introducing either a aMTJ or TJ separately (2s-MTJ, 2s-TJ) increases cost of transport (CoT) by 7–12% compared to the rigid model (1s-lA). Coupling arch and toe joint motion (3s-W) results in a similar CoT as for rigid models, which is in contrast to^21^ where models with a windlass effect also had increased CoT. Adding a toe consistently decreases stride length by up to 7%, increases stride time by up to 19%, and decreases HAT velocity by up to 21% compared to the fastest model, 2s-MTJ. These trends are independent of whether the TJ is coupled to the MTJ or not. Positive ankle power in late stance correlates with walking speed in humans^50^, i.e. higher walking speed is associated with higher positive power and vice versa. This is also reflected in our results, as the fastest model (2s-MTJ) has the highest peak ankle power, see Fig. 5b.

**Table 4:**
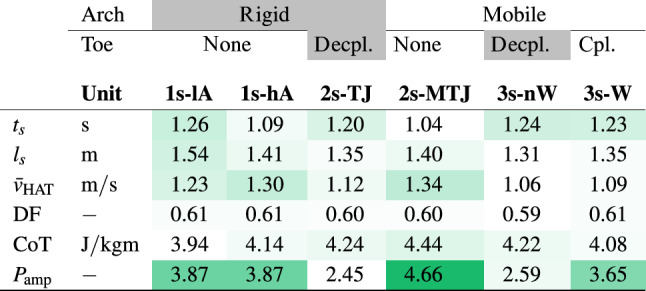
Global gait measures for all models.

The table includes stride time $t_s$, stride length $l_s$, mean velocity of upper body segment $\bar{v}_{HAT}$, duty factor $DF = \frac {t_\text {stance}}{t_\text {stride}}$, CoT $= \frac {E_{\text {metab}}}{m \cdot l_\text {s}}$ and power amplification according to^28^ $P_{\text {amp}} = \frac {|\text {max}(P_{\text {ank}}|P_{\text {ank}}>0)|}{|\text {min}(P_{\text {ank}}|P_{\text {ank}}<0)|}$. The intensity of the color $I_\text {col}$ for each cell shows its deviation from the minimum value within the row of the table $x_\text {row, min}$ with: $I_\text {col} = \frac {(x_\text {cell} - x_\text {row, min})}{x_\text {row, min}} \in [0, 1]$. CoT results compare well with literature:^17^reported a 7–8% higher CoT of 4.22 J/kgm for models with TJ compared to models with rigid feet.

### Internal foot kinematics

Figure 6 shows the kinematics of MTJ and TJ. The human range of motion is only approximated when TJ and MTJ are coupled (3s-W). All other models show insufficient arch mobility between 50 and 60% stride where the MTJ is plantar-flexing, shortening the arch while dorsi-flexing the toe. If TJ and MTJ are not coupled (3s-nW), arch shortening is not possible because PF and PL have their neutral position at $0^{\circ }$ MTJ angle. The maximum toe flexion of $35^{\circ }$ for decoupled toe models (2s-TJ, 3s-nW) is half that of the coupled model at $68^{\circ }$, where the shortening of the arch allows more toe movement. The toe resting angle during swing is between $20^{\circ }$ and $30^{\circ }$ flexion in humans^33,51^ while being $0^{\circ }$ for the simulation. This is because our model has no active muscles to lift the toe during swing. The maximum PF elongation is between 1.0% and 1.3% of $l_0$, comparable to literature^52^.

## Discussion

Our goal was to first answer the question **(I) What is the effect of a mobile arch, a toe joint, and the coupling of toe and arch motion through the PF on gait prediction for neuromuscular simulations?** We focused on GRFs, energy dissipation during contact, joint kinematics for the hip, knee, and ankle, and ankle push-off behavior as a key element of walking efficiency, walking speed, and natural leg dynamics.

Adding a TJ reduces the first GRF peak and shifts the second vertical GRF peak earlier and while maintaining similar hip, knee, and ankle stance kinematics compared to rigid foot models. Around push-off, the propulsive horizontal GRFs are reduced and the ankle power amplification decreases by 50% compared to the model with the highest power peak (2s-TJ vs. 2s-MTJ). The reduced push-off is associated with a less dynamic gait, as evidenced by increased stride time and CoT, as well as reduced forward walking velocity and stride length. Similar findings were shown on lower limb prostheses with TJs of different stiffness^53^ or fixed versus mobile TJs^54^.

A mobile arch allows for improved forward propulsion and push-off capabilities, as seen in the power amplification ratios in Table 4. Although the simulated arch recoil for 2s-MTJ shown in Fig. 6 is low compared to literature, we still observe that the presence of the arch has a beneficial effect on push-off. This is consistent with the results of a recent study using radiographic measurements of foot bone motion during walking, which showed that a mobile arch helps to improve forward propulsion^55^.

The coupling of TJ and MTJ mitigates the negative effects of adding a toe, and the joint kinematics for TJ and MTJ are comparable to human data. The fully coupled model (3s-W) achieves similar performance to rigid models in terms of ankle push-off power and energy efficiency. Only the walking speed remains significantly slower. The coupling of arch and toe motion appears to be essential for ankle push-off through power transfer between the TJ and the MTJ, as previously shown by^49,56^. When the TJ stretches the PF during push-off, the induced tension produces positive work in the arch of the foot, whereas in the absence of coupling, the TJ only dissipates energy.

Second, we answer the question based on our findings: **(II) How much of the foot’s anatomy do we need to model to predict sagittal plane walking kinematics and kinetics with neuromuscular simulations in good agreement with human data?** We found no significant differences in knee, hip, and ankle stance kinematics for all models. The rigid models achieved similar walking speeds and ankle power amplification compared to our most complex model, 3s-W. Therefore, if the main interest of a study is not the foot itself, a simpler model may be sufficient. Only when details of ankle kinematics and internal foot motion at MTJ and TJ are relevant one should choose a model that includes joint coupling between TJ and MTJ to mimic human foot kinematics and global walking behavior.

Foot and arch stiffness are difficult to define because arch stiffness is not uniform throughout the gait cycle and different studies differ in their measurements of effective stiffness and damping properties^30,52,57^. We chose the modeling approach of^30^, using their measured properties for PF, and calibrated the cross-sectional area and damping for the PL under static loading conditions by simulating a test bench from^23^, see supplementary material Table S2 for details. All our models are over-damped and slightly stiffer compared to human data. For dynamic motion, ligament stiffness and damping may be different from what we obtained in our procedure. In particular, very soft ligaments would likely reduce ankle push-off^20^. Therefore, we tested our models with half of the default cross-sectional ligament areas for PL and PF, thus reducing the effective stiffness and damping. The trends described for GRFs, joint kinematics, ankle push-off, and energetics, i.e. the key messages of this paper, still hold.

Farris et al.^58^ demonstrated that muscle contractions play a role in stiffening the foot during push-off, and simulations also suggest that active muscle stiffening in the foot may be essential for push-off^20^, altering the behavior of the plantarflexor muscles^59^. Muscles also appear to alter the energetic behavior of the foot^60^. Our models do not have active muscles and therefore cannot actively stiffen at the end of stance. However, the joint work for the MTJ and TJ was comparable to that obtained in^20^. Our investigations suggest that active foot stiffening is not necessary to achieve impulsive ankle push-off, at least for walking. Maintaining a constant stiffness throughout the gait cycle, as seen in fully rigid models, also leads to impulsive ankle push-off, provided the foot is “sufficiently stiff”. This aligns with recent studies in biology that demonstrate that the (quasi-)stiffness of the human foot does not increase before or during propulsion^61^. The specific mechanisms involved in human walking are still unknown. However, for our simulations, the coupling between MTJ and TJ through PF seems to be a determining factor. The windlass effect actively shortens the arch, resulting in an altered shape and improved energy efficiency of the expected gait^23^.

We use a simple, generic 2D model with 14 muscles and a local reflex-based controller^4^. Since we analyze normal walking without perturbations or impairments, we consider 2D simulations sufficient. The primary axes of motion for normal walking at the knee, hip, and ankle joints^48^, ankle push-off^28^, and toe motion occur in the sagittal plane. In addition, ground reaction forces in the vertical and anterior-posterior directions are one to two orders of magnitude higher than in the mediolateral direction^48^. However, for other applications, such as balance, 3D simulations are required^62^.

The reflex controller is based on several principles of locomotion^4^ that are encoded in the reflex architecture, thereby reducing the parameter space for optimization^11,63^. Only the feedback gains need to be optimized to achieve steady-state walking without the need for subject-specific measurements, but the predefined control architecture also has some drawbacks. The way the controller implements the load transfer between legs^4^, Fig. 1e and section C, p. 264f and the stabilization of the upper body^4^, Fig. 1d and Section B, p. 264f induces artifacts in the results where the simulation differs significantly from human data, regardless of the foot model chosen. All models show early heel-off, premature ankle unloading, and differences in ankle-knee coordination during push-off. The peak of dorsiflexion in the human data occurs around opposite touch-down, while the model has its dorsiflexion peak around heel-off, 20% earlier in the gait cycle, as shown in Fig. 5b. Push-off in human gait begins when the knee begins to flex and the ankle begins to plantarflex^28^. In the models, the timing is compromised because the peak of ankle dorsiflexion occurs too early. The second GRF peak is low in all models, and the upper body is tilted forward.

The results for ankle push-off may vary when using different approaches^7^ that are not limited to a specific reflex architecture. Future work could apply our foot models in other simulation environments, such as open-loop control, which is possible with OpenSim Moco^64^.

The cost function we used to optimize the steady-state walking models has a large influence on the predicted walking patterns^6,7,17^. Our cost function includes only basic locomotor primitives such as metabolic energy reduction^65^. We rely on the assumption that if the mechanical model design is well suited for its purpose of walking, a natural gait will emerge from simple control primitives^39^ without the need for explicitly tracking joint kinematics and kinetics in the cost function. All optimizations converged, but we cannot guarantee that our solutions are the global minimum for the chosen cost function. Since we are using a multi-objective cost function, changes in weighting of the individual contributions and the addition of more constraints could lead to different results. To differentiate the effects of optimization and foot model, we also analyzed the behavior of all models using non-optimized control parameters from^4^ shown in the supplementary material, Figs. S1–S6 and Table S3. Our results presented in this paper were consistently observed also without optimization. The TJ and MTJ kinematics were independent of optimization, as was the ankle push-off kinematics. Conversely, optimization significantly affected the knee and hip kinematics, while the foot model had no significant effect.

GRFs are strongly influenced by the mathematical contact formulation and related parameters such as contact stiffness, damping properties, and numerical aspects such as step size and solver selection. We have used the same formulation for all models and have not investigated the mathematical contact formulation in detail. However, we would like to emphasize that computational efficiency and simulation accuracy are highly dependent on it.

## Conclusion

How the foot is modeled for generic, reflex-controlled predictive neuromuscular simulations widely used in research, e.g. with the open-source software SCONE^1^, affects gait prediction. In particular, ankle push-off and ankle kinematics during terminal stance are affected by the presence of a mobile arch and/or toe versus a single rigid segment. However, for many research purposes, simpler, rigid foot models may be sufficient, which is in line with findings for subject-specific simulations^66^. Model complexity increases with the number of segments, and more modeling assumptions must be made that are subject to uncertainty and may have an unclear effect on the results^67^. For this reason, a biomechanical model should always be tailored to its specific purpose. A rigid foot model is sufficient for investigating the joint kinematics of the ankle, knee, and hip without focusing specifically on the foot. The choice of cost function for optimization is likely to have a more significant impact on the simulation results than the design of the foot^6^. When studying ankle push-off behavior or analyzing foot details such as TJ or arch motion, foot pathologies or walking on uneven surfaces^68^, a more complex model is beneficial. For inner foot joint kinematics, a model with a mobile arch and coupled TJ should be used to resemble human foot kinematics. Adding only one toe to a rigid arch worsens the ankle’s push-off power, resulting in slower and less efficient gait. A flexible arch without a toe is advantageous for dynamic walking with a higher peak ankle push-off power.

### Supplementary Information

Supplementary Information.

## Data Availability

All data and code related to this manuscript are available in the following GitHub repository: https://gitlab.lrz.de/ecowalk/foot-ground-contact-modeling. Human data from van der Zee et al.^32^ that we used for comparison are available at: https://github.com/timvanderzee/human-walking-biomechanics.
