# Supplementary material for: The effect of including a mobile arch, toe joint, and joint coupling on predictive neuromuscular simulations of human walking
Source: Sci Rep. 2024 Jun 27;14:14879. doi: 10.1038/s41598-024-65258-z (PMC11211509; doi:10.1038/s41598-024-65258-z)
Supplement: Supplementary file 1 — Supplementary Information. [file 41598_2024_65258_MOESM1_ESM.pdf]

Supplementary material for *"The effect of including a mobile arch, toe joint, and joint coupling on predictive neuromuscular simulations of human walking"*

Alexandra Buchmann, Simon Wenzler, Lauren Welte and Daniel Renjewski

S1 Cross correlation

Table S1: Cross correlation to human data from [32] for hip, knee and ankle kinematics during swing and stance phase. An R-value of one indicates perfect alignment with reference data, zero shows that the signals are not similar at all.

Arch	rigid			mobile		
Toe	none		decpl.	none	decpl.	cpl.
	**1s-lA**	**1s-hA**	**2s-TJ**	**2s-MTJ**	**3s-nW**	**3s-W**
$R_{\text{ank, stance}}$	0.82	0.90	0.85	0.86	0.81	0.68
$R_{\text{ank, swing}}$	0.79	0.81	0.17	0.17	0.73	0.70
$R_{\text{kne, stance}}$	0.97	0.99	0.99	0.98	0.98	0.99
$R_{\text{kne, swing}}$	0.99	0.99	0.99	0.99	0.98	0.98
$R_{\text{hip, stance}}$	0.93	0.97	0.96	0.96	0.94	0.94
$R_{\text{hip, swing}}$	0.99	0.99	0.98	0.99	0.96	0.96

S2 Foot deformation under static loading conditions

Table S2: Comparison of human data from [23, Table 2] and our virtual test bench. The simulation data were obtained using the fast loading condition. In [23], the arch elongation was calculated using $(l_{\text{max}}-l_{\text{min}})/l_{\text{max}}$. We normalized all values to the mean of the maximum arch lengths of 0.2601 m [23]. We set the static load to 1.0 times the body weight.

		plantarflexed		dorsiflexed	
	**Unit**	**Simulation**	**Human [23]**	**Simulation**	**Human [23]**
arch elongation	—	5.4	5.6 ± 2.3	12.1	8.7 ± 2.9
$\Delta\varphi_{\text{MTJ}}$	deg	1.8	2.9 ± 0.8	3.7	2.6 ± 1.0
energy absorbed	mJ/kg	34.7	22.5 ± 5.8	38.8	25.9 ± 9.9
energy returned	mJ/kg	11.7	16.7 ± 5.5	17.2	17.9 ± 5.9
energy dissipated	mJ/kg	23.0	5.9 ± 4.0	21.7	8.0 ± 5.9
energy ratio	—	0.34	0.74 ± 0.15	0.44	0.71 ± 0.12

S3 Optimized versus non optimized solutions

Ground reaction forces (fig. S1) and foot model work (fig. S2) The cost function for the optimization includes reducing the GRF rates, see Equation (4). This reduces the GRFs and the dissipative work done by the contact elements for all optimized models as expected. General trends described in the results, such as the influence of adding a TJ on the horizontal GRFs during push-off, are independent of optimization. For a mobile arch without toe (2s-MTJ), the first GRF hump is higher in the optimized solution and significantly different from all other models. This may be due to a local minimum of the optimization.

Joint kinematics (fig. S3), ankle push-off (fig. S4) and SOL & GAS behavior (fig. S5) Changes in ankle plantarflexion have almost no effect on push-off behavior because most of the increased plantarflexion occurs after toe-off. The increased plantarflexion at the end of stance is unlikely to be due to specific morphological changes in the foot models, but rather is an artifact of the optimization. Adding decoupled toes to a rigid or mobile arch reduces plantarflexion, while fully rigid models and coupled MTJ and TJ models result in increased plantarflexion.

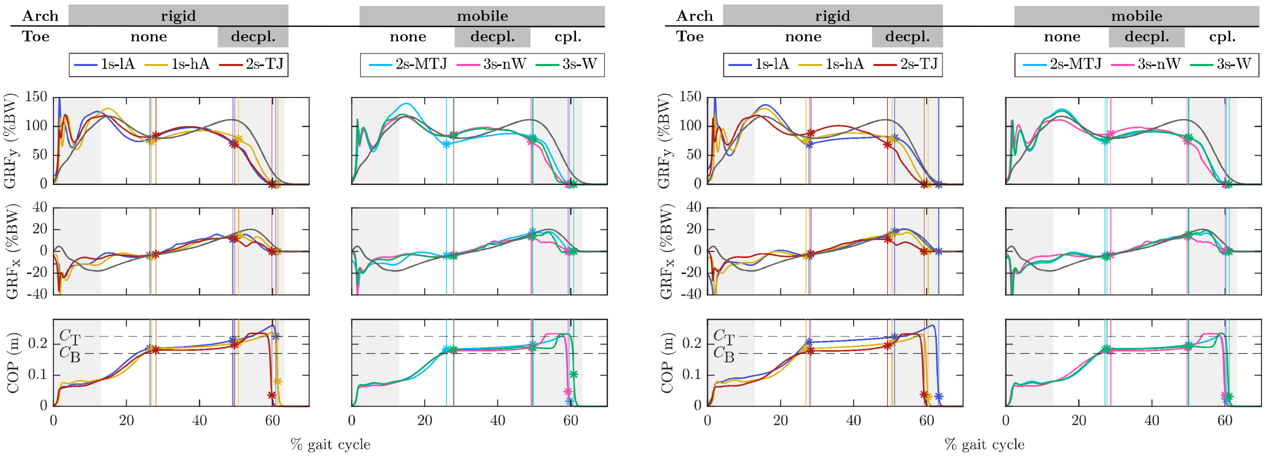

(a) Optimized Control Parameters.

(b) Not optimized, default [4] control parameters.

Figure S1: Vertical and horizontal GRFs and CoP progression for optimized versus non-optimized solutions for all foot models. Light gray areas indicate double support for human data [48]. Vertical colored lines indicate heel-off, opposite leg's touchdown and toe-off for each model. C_B and C_T indicate the positions of the ball and toe contact elements (see fig. 3).

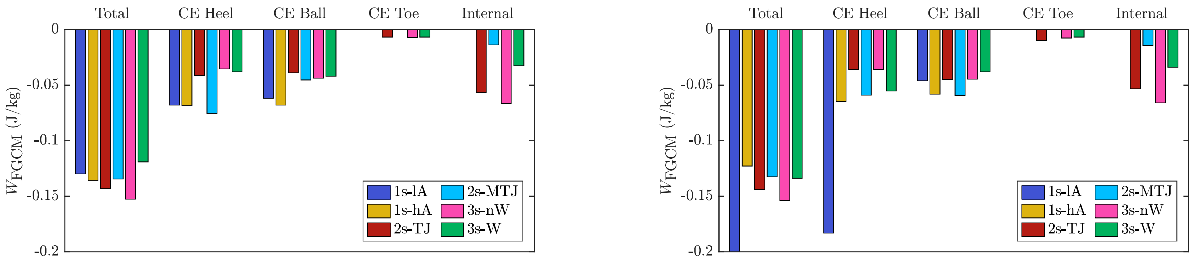

(a) Optimized Control Parameters.

(b) Not optimized, default [4] control parameters.

Figure S2: Foot model work in total and divided into contact element and ligaments for optimized versus non-optimized solutions. The rigid arch model with low ankle shows large differences before and after optimization, as the model was not able to walk stably with the default control parameters from [4].

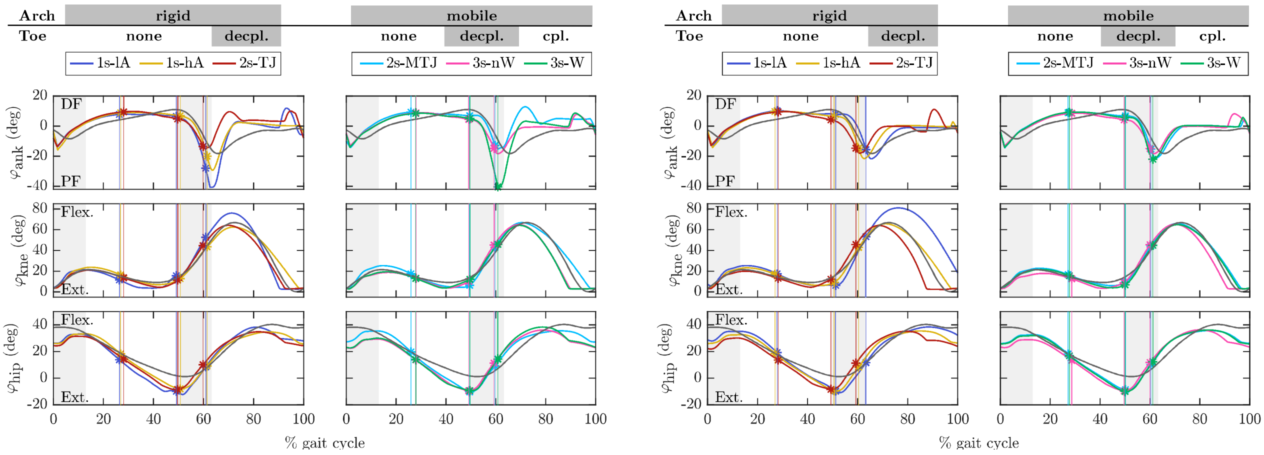

(a) Optimized Control Parameters.

(b) Not optimized, default [4] control parameters.

Figure S3: Joint kinematics for ankle, knee and hip for optimized versus non-optimized solutions. Directions of motion are shown for flexion (Flex.), extension (Ext.), dorsiflexion (DF), and plantarflexion (PF). Optimization shows a large effect on ankle plantarflexion around push-off for rigid arch models without toe as well as for mobile arch coupled with TJ motion. Plantarflexion after push-off is substantially increased from 20 deg to 40 deg. The hip is not influenced, the knee timing is closer to human data.

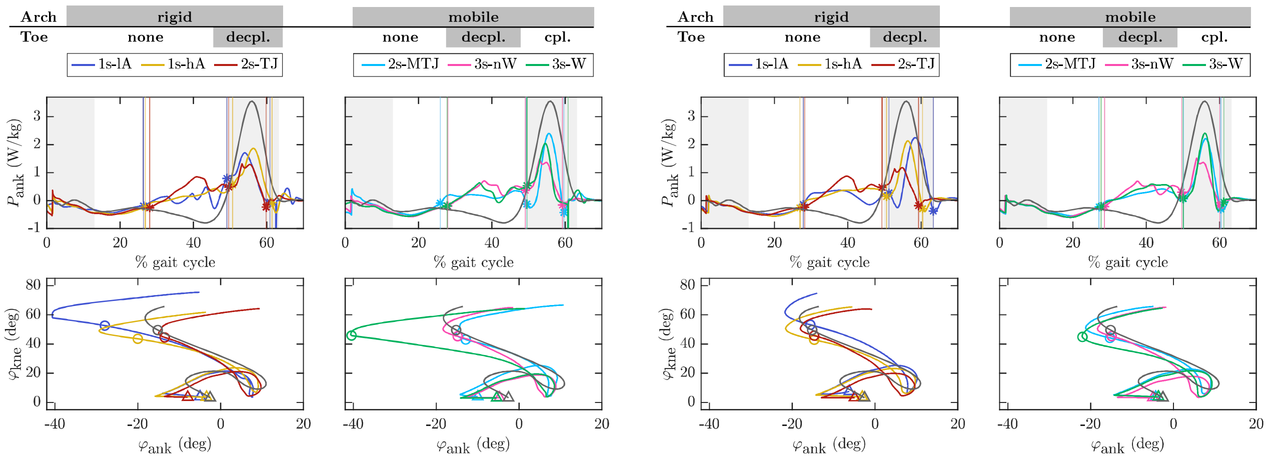

(a) Optimized Control Parameters.

(b) Not optimized, default [4] control parameters.

Figure S4: Ankle power and knee-ankle coordination for optimized versus non-optimized solutions. Although the ankle plantarflexion changes significantly for optimized versus non-optimized solutions, the resulting ankle push-off behavior does not change as much. General trends such as reduced push-off performance for models with decoupled TJ and rigid arch are maintained throughout the optimization process.

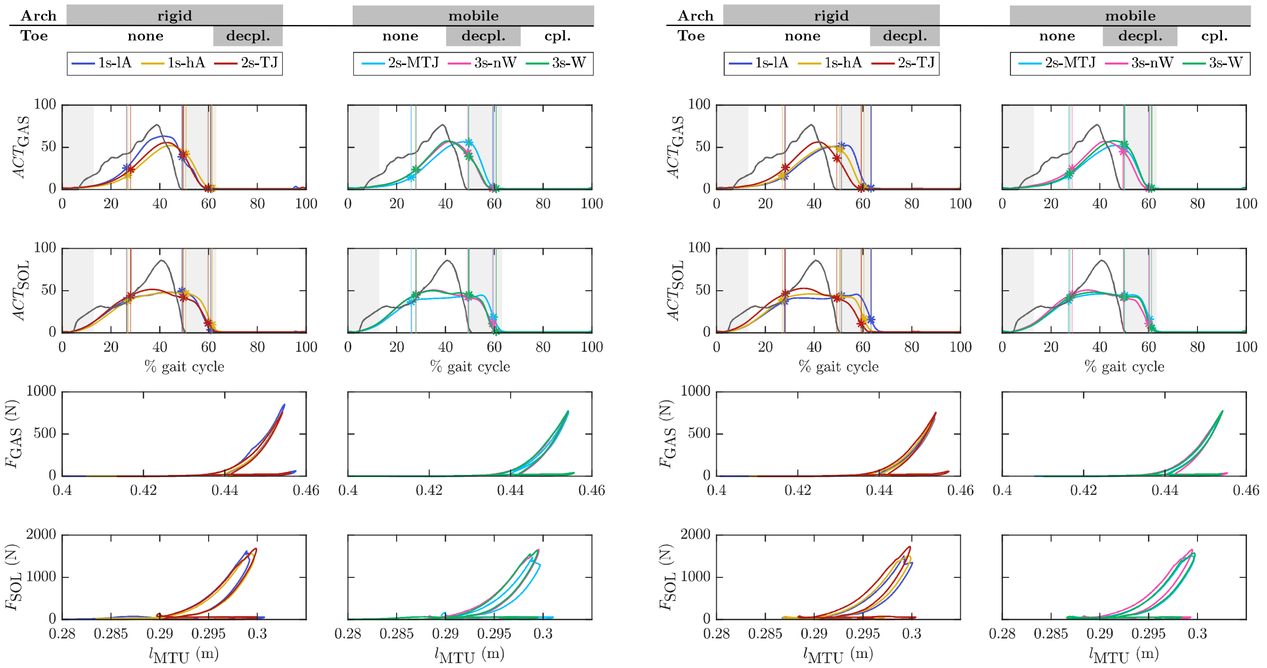

(a) Optimized Control Parameters.

(b) Not optimized, default [4] control parameters.

Figure S5: Force-length curves and activation for plantar flexor muscles SOL and GAS for optimized versus non-optimized solutions. GAS and SOL are active until the end of stance in the model, while human muscles are only active until the opposite touchdown. Inclusion of MTJ reduces SOL activation in the second half of stance, but increases GAS activation. 2s-MTJ has a slower SOL contraction velocity, i.e. a less steep force-length curve. Human data from [48].

Internal Foot Kinematics of MTJ and TJ (fig. S6) The internal foot kinematics is determined by the properties of PF and PL as well as the presence of a joint coupling between TJ and MTJ. Both are not part of the optimization process, i.e. the motion is not affected. The change in MTJ motion for the fully coupled model is the result of increased ankle plantarflexion reducing MTJ plantarflexion.

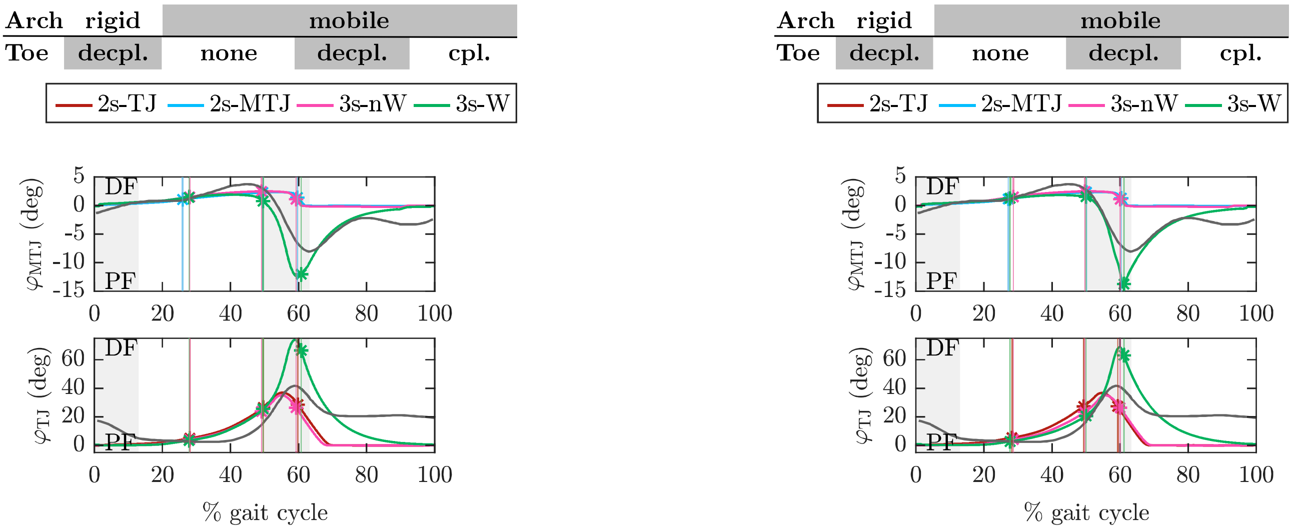

(a) Optimized Control Parameters.

(b) Not optimized, default [4] control parameters.

Figure S6: Joint kinematics for TJ and MTJ for optimized versus non-optimized solutions. Gray lines show human data from [33]. Both, MTJ and TJ kinematics are not affected by the optimization process. The MTJ motion becomes slightly smoother and reduced in amplitude for the fully coupled model (3s-W) around toe-off for the optimized solution.

Global Gait Measures General trends described in the results, e.g. slower walking for models with toe, and highest CoT for 2s-MTJ, are maintained throughout the optimization process. The non-optimized solution for 1s-lA should be treated with caution, as the model is not able to walk stably. Overall, the optimization appears to make only minor adjustments to the global model behavior.

Table S3: Global gait measures for all models with (left) and without (right) optimization. The tables show stride time t_s, stride length l_s, average upper body segment velocity v_{HAT}, duty factor DF, and CoT for both, and power amplification P_{amp} for the optimized solution only.

Arch		rigid			mobile		
Toe		none		decpl.	none	decpl.	cpl.
	**Unit**	**1s-lA**	**1s-hA**	**2s-TJ**	**2s-MTJ**	**3s-nW**	**3s-W**
t_s	s	1.26	1.09	1.20	1.04	1.24	1.23
l_s	m	1.54	1.41	1.35	1.40	1.31	1.35
$\bar{v}_{\text{HAT}}$	m/s	1.23	1.30	1.12	1.34	1.06	1.09
DF	—	0.61	0.61	0.60	0.60	0.59	0.61
CoT	J/kgm	3.94	4.14	4.24	4.44	4.22	4.08
P_{amp}	—	3.87	3.87	2.45	4.66	2.59	3.65

Arch		rigid			mobile		
Toe		none		decpl.	none	decpl.	cpl.
	**Unit**	**1s-lA**	**1s-hA**	**2s-TJ**	**2s-MTJ**	**3s-nW**	**3s-W**
t_s	s	0.98	1.05	1.27	1.06	1.21	1.08
l_s	m	1.35	1.40	1.32	1.36	1.31	1.34
$\bar{v}_{\text{HAT}}$	m/s	1.37	1.33	1.04	1.28	1.08	1.23
DF	—	0.63	0.60	0.59	0.60	0.60	0.61
CoT	J/kgm	5.12	4.29	4.21	4.38	4.26	4.37
